# Iterative sub-network component analysis enables reconstruction of large scale genetic networks

**DOI:** 10.1186/s12859-015-0768-9

**Published:** 2015-11-04

**Authors:** Naresh Doni Jayavelu, Lasse S. Aasgaard, Nadav Bar

**Affiliations:** Department of Chemical Engineering, Norwegian University of Science and Technology (NTNU), Sem Salandsvei 4, Trondheim, Norway

**Keywords:** Network analysis, Gene expression analysis, Iterative method, Partial least square, Gene regulation, Dynamic modeling

## Abstract

**Background:**

Network component analysis (NCA) became a popular tool to understand complex regulatory networks. The method uses high-throughput gene expression data and a priori topology to reconstruct transcription factor activity profiles. Current NCA algorithms are constrained by several conditions posed on the network topology, to guarantee unique reconstruction (termed compliancy). However, the restrictions these conditions pose are not necessarily true from biological perspective and they force network size reduction, pruning potentially important components.

**Results:**

To address this, we developed a novel, Iterative Sub-Network Component Analysis (ISNCA) for reconstructing networks at any size. By dividing the initial network into smaller, compliant subnetworks, the algorithm first predicts the reconstruction of each subntework using standard NCA algorithms. It then subtracts from the reconstruction the contribution of the shared components from the other subnetwork. We tested the ISNCA on real, large datasets using various NCA algorithms. The size of the networks we tested and the accuracy of the reconstruction increased significantly. Importantly, FOXA1, ATF2, ATF3 and many other known key regulators in breast cancer could not be incorporated by any NCA algorithm because of the necessary conditions. However, their temporal activities could be reconstructed by our algorithm, and therefore their involvement in breast cancer could be analyzed.

**Conclusions:**

Our framework enables reconstruction of large gene expression data networks, without reducing their size or pruning potentially important components, and at the same time rendering the results more biological plausible. Our ISNCA method is not only suitable for prediction of key regulators in cancer studies, but it can be applied to any high-throughput gene expression data.

**Electronic supplementary material:**

The online version of this article (doi:10.1186/s12859-015-0768-9) contains supplementary material, which is available to authorized users.

## Background

Gene expression is a highly regulated process and difficult to understand without computer added tools. The relationship between target genes (TG) and their regulators, the transcription factors (TF), is complex and even simple gene expression studies usually incorporate hundreds of TGs, TFs and the relationship between them. Several statistical methods including principal component analysis (PCA), singular value decomposition (SVD), independent component analysis (ICA), partial least squares regression (PLSR) and their variants were successfully applied on expression data to extract biologically significant knowledge [1–4]. However, these methods incorporate statistical assumptions, either of orthogonality and/or statistical independence which are not true for biological data [5]. Network component analysis (NCA) attempts to overcome these limitations [6]. The NCA integrates gene expression and a priori TF-TG connectivity data (known relationships obtained from previous experiments) and computes the activities of the TFs and the connectivity strength of each TF to their TGs. The decomposition of the gene expression matrix (termed *E*) into a topology (termed *A*, relating the observed TF and TG expression covariance patterns) and a temporal score matrix (termed *P*, describing the TF activity development pattern), according to model:

(1) $$ E=AP + \epsilon  $$

This is achieved by solving a bilinear least squares optimization problem. In order to guarantee a unique solution up to scale, the matrices *A* and *P* are subjected to three conditions, termed as NCA criteria (see ‘Methods’) [6]. Briefly speaking, the first condition implies that there cannot be two or more TFs with the same regulatory functionality. This makes little sense, because it is well known that redundancy is very common in living systems, as it contributes to robustness [7]. Another condition implies that there cannot be two or more TFs or TF combinations with the same temporal behavior, but again it is not consistent with our knowledge that TFs often work cooperatively [8, 9]. Therefore, these conditions imply restrictions that do not seem plausible from biological perspective. Moreover, these conditions pose necessary restrictions on the size and structure of the network [6], and the problem with the current solutions is that in order to avoid false discovery (outcome of non-unique solutions), they usually reduce the size of the network significantly, losing in the process potentially important components. Therefore, we seek to avoid these restrictions if possible.

The original NCA algorithm suffered from unstable solutions due to ill-conditioned matrices and multiple local solutions. Tikhonov regularization method (termed as GNCA-r) overcomes these two issues but is computationally expensive for solving larger networks [10]. Fast network component analysis (FastNCA) is a stable and fast approach, up to several hundred times faster than GNCA-r but limited to smaller networks [11]. Recently, the robust network component analysis (ROBNCA) was developed that offers a stable, efficient and accurate solution, by explicitly modeling the presence of outliers in the microarray data [12]. Whereas these approaches were focused primarily on improving the accuracy of reconstruction, they were all subjected to the same (limiting) criteria mentioned above, that force reduction of the network size. The issues of limited network size and removal of key TFs from the network to satisfy the NCA conditions were the focus of several research groups [10–16]. For instance, the division of large networks into smaller, overlapping NCA compliant ones helped to reconstruct some of the shared components. However, this approach treated the sub-networks independently, as if they were obtained from different datasets. It ignores the inter-connections exist between the sub-networks. More specifically, when computing the least square of one sub-network using this method, the contributions of the shared TGs and TFs from all the other sub-networks are ignored, consequentially loosing valuable information. It is a heuristic approach and works only for specific network configurations, but does not work for the general case [13].

We propose a novel algorithm, termed Iterative Sub-Network Component Analysis (ISNCA), which solves compliant sub-networks, and iterates between them in order to provide a solution to the complete, possible incompliant, network. The ISNCA predicts a solution using a standard NCA algorithm on one sub-network to update the common components in the expression matrix of the other. Then the ISNCA predicts the solution of the other sub-network (using the same standard NCA algorithm), in order to update the first one. This is done iteratively until the error reconstruction of the entire network (see ‘Methods’) convergences to a minimum. We tested first the performance of the ISNCA algorithm against the common GNCA-r [10] for a small synthetic network that is compliant (i.e. satisfying the three necessary conditions). Secondly, we compared the performance of ISNCA iterating on a small, synthetic, incompliant network that was divided into two compliant sub-networks. We applied the ISNCA using GNCA-r, FastNCA [11] and ROBNCA [12], to solve the entire network in an iterative manner. We compared also the stability of the iterations and the accuracy of the complete network solution. Finally we tested our proposed algorithm on two, full scale, independent, real biological expression data, each containing hundreds of genes with more than 200 network configurations. We compared the solutions of the ISNCA to standard NCA algorithms, and showed that our proposed method retains many essential components in breast cancer studies, that otherwise were removed by standard NCA.

## Methods

### Network component analysis algorithms

Network component analysis algorithms decompose gene expression data matrix into a weighted topology TF-TG matrix and the temporal profile matrix of the TFs. The model can be represented in the matrix form as follows:

(2) $$ E=AP + \epsilon  $$

where, $E\in \mathbb {R}^{n\times m}$ represents an expression matrix, $A\in \mathbb {R}^{n\times l}$ represents the initial connectivity matrix, defining the sign and size of how each of the *n* target genes involved in this network are linked to each of the *l* transcription factors involved, in terms of *l* regulatory patterns. $P\in \mathbb {R}^{l\times m}$ represents the TF activity matrix, defining how each of the *l* regulatory transcription factor pattern develops over time. The index *m* is the number of time points or measurement conditions. The decomposition of *E* into *A* and *P* is achieved by solving a bilinear alternating least squares optimization problem subjected to three conditions termed as NCA criteria: (i) the connectivity matrix *A* should be full-column rank; this means that each of the *l* transcription factor patterns in this network contribute some unique variation, so that the number of independent transcription factor patterns equals the number of TFs included. Otherwise they may be difficult to observe experimentally. (ii) If a column is removed from *A* as well as TGs connected to it, the resulting matrix still should be full-column rank; (iii) TF activity matrix *P* should be full-row rank, which means that the temporal behavior of each of the *l* regulatory patterns should have different kinetics - otherwise they cannot be distinguished experimentally.

### Iterative sub-network component analysis (ISNCA)

**Fig. 1:**
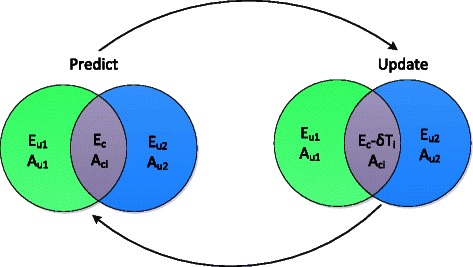
Graphical representation of ISNCA approach. ISNCA approach involves predict and update phases. Subscripts *u*_*i*_ and *c*_*i*_ represent unique and common components of subnetwork *i*, respectively. See ‘Methods’ for the formulations of *A*, *E*, and *T*

We propose a novel algorithm, the iterative sub-network component analysis (ISNCA), that iterates between NCA compliant, overlapping sub-networks (Fig. 1). These sub-networks share common TGs in order to solve larger, and most importantly, NCA incompliant networks. In order to apply the ISNCA, we first divide the network into two compliant sub-networks. The expression and connectivity matrices for each sub-network can be represented by

(3) $$ E_{1}=\left[\begin{array}{ll} E_{u1} \\ E_{c} \end{array}\right], E_{2}=\left[\begin{array}{ll} E_{u2} \\ E_{c} \end{array}\right]  $$

and

(4) $$ A_{1}=\left[\begin{array}{ll} A_{u1} \\ A_{c1} \end{array}\right], A_{2}=\left[\begin{array}{ll} A_{u2} \\ A_{c2} \end{array}\right]  $$

with $E_{\textit {ui}}\in \mathbb {R}^{nui\times m}$ and $E_{c}\in \mathbb {R}^{nc\times m}$ denote the expression matrices of sub-networks *i*=1,2, the index *c* denotes the common components, *ui* are the unique components of sub-network *i*, and $A_{\textit {ui}}\in \mathbb {R}^{nui\times lui} A_{\textit {ci}}\in \mathbb {R}^{nc\times lui}$ are the partition matrices of *A*. Assuming no TFs are shared between the networks, the decomposition of *P* is simply $P_{i}=P_{\textit {ui}}\in \mathbb {R}^{lui\times m}$. A graphical representation of the approach is shown in Fig. 1. In all the following, when we write *A*_*i*_, *E*_*i*_ or *P*_*i*_, we refer to matrices of the entire sub-network *i*, including both its exclusive and common components.

The entire network can be described in the following manner:

(5) $$ A=\left[\begin{array}{ll} A_{u1} & \mathbf{O}_{2} \\ \mathbf{O}_{1} & A_{u2} \\ A_{c1} & A_{c2} \end{array}\right]  $$

The matrices $\mathbf O_{1}\in \mathbb {R}^{nu2\times lu1}$ and $\mathbf O_{2}\in \mathbb {R}^{nu1\times lu2}$ denote zero matrices. Assuming that *P* does not have common components, the corresponding partitions of *E* and *P* can be obtained as follows:

(6) $$ E=\left[\begin{array}{ll} E_{u1} \\ E_{u2} \\ E_{c} \end{array}\right], \qquad P=\left[\begin{array}{ll} P_{u1} \\ P_{u2} \end{array}\right]  $$

where $P_{\textit {ui}}\in \mathbb {R}^{lui\times m}$ are the activities of the unique TFs of sub-network *i*.

#### Example 1.

Network decomposition: Consider the network presented in Case Study 2 (Fig. 3 a). The connectivity matrix *A* can be decomposed to the exclusive components and the common components in the following manner:
(7)


and partition matrices for sub-networks 1 and 2 respectively are,

$$ A_{u1}=\left[\begin{array}{ll} 1 & 1 \\ 1 & 0 \\ 0 & 1 \end{array}\right], \qquad \mathbf{O}_{1}=\left[\begin{array}{ll} 0 & 0 \end{array}\right], \qquad A_{c1}=\left[\begin{array}{ll} 1 & 0 \\ 0 & 1 \\ 0 & 1 \end{array}\right] $$

$$ A_{u2}=\left[\begin{array}{ll} 1 & 0 \end{array}\right], \qquad \mathbf{O}_{2}=\left[\begin{array}{ll} 0 & 0 \\ 0 & 0 \\ 0 & 0 \end{array}\right], \qquad A_{c2}=\left[\begin{array}{ll} 1 & 1 \\ 1 & 0 \\ 0 & 1 \end{array}\right] $$

To initialize the ISNCA algorithm, we divide the expression matrix, *E* to *E*_*i*_ using Eq. 3 and connectivity matrix, *A* to *A*_*i*_ using Eq. 4. At the start of each iteration *k*, we compute solution to ∥*E*_*i*_(*k*)−*A*_*i*_ *P*_*i*_∥, separately for sub-networks 1 and 2 using any standard NCA method, and obtain $\hat A_{i}(k)$ and $\hat P_{\textit {ui}}(k)$. We can then proceed to construct $\hat A(k)$ and $\hat P(k)$ by combining Eqs. 4 and 5, as

(8) $$ \hat A(k)=\left[\begin{array}{ll} \hat A_{u1}(k) & \mathbf{O}_{1} \\ \mathbf{O}_{2} & \hat A_{u2}(k) \\ \hat A_{c1}(k) & \hat A_{c2}(k) \end{array}\right], \hat P(k)=\left[\begin{array}{ll} \hat P_{u1}(k) \\ \hat P_{u2}(k) \end{array}\right]  $$

and calculate the error of the entire network,

(9) $$ e(k)=\big\|{E-\hat A \hat P}\big\|_{F}  $$

If the error does not converge (see below), we proceed to update the sub-networks in the following manner. Let *T*_*i*_(*k*) be the common TGs contribution from sub-networks *i*, that is,

(10) $$ T_{1}(k)=\hat A_{c1}(k)\hat P_{u1}(k), \qquad T_{2}(k)=\hat A_{c2}(k)\hat P_{u2}(k)  $$

We then update the matrices *E*_1_ and *E*_2_ for next iteration, from Eq. 11 by subtracting the common TGs contribution from other sub-network, that is,

(11) $$\begin{array}{*{20}l} E_{1}(k+1)&=\left[\begin{array}{ll} E_{u1} \\ E_{c}-\delta\cdot T_{2}(k) \end{array}\right],\\ E_{2}(k+1)&=\left[\begin{array}{ll} E_{u2} \\ E_{c}-\delta\cdot T_{1}(k) \end{array}\right] \end{array} $$

Here, *δ*∈ [0,1] denotes the attenuation factor (see below for details). Notice that *E*_*c*_ and *E*_*ui*_ do not change from iteration to iteration as they represent the original expression matrices. We then proceed to the next iteration and predict the solution to the expression ∥*E*_*i*_(*k*)−*A*_*i*_ *P*_*i*_∥ using standard NCA methods. We keep iterating until the reconstruction error in Eq. 9 for the entire network is sufficiently small, for instance by

(12) $$ e(k+1)-e(k)<\epsilon  $$

In our simulations, we set *ε* to be 1e-05 and maximum number of iterations to 100.



### Microarray data and preprocessing

The microarray data used in this case study was obtained by treating the MCF7 breast cancer cells with two growth factors (Epidermal growth factor, EGF and Heregulin, HRG) at different time points over a period of 0–72 hours [17]. We downloaded the data from GEO data base with array express accession number: GSE13009. We applied loess normalization within time points and quantile normalization across time points. The expression values were averaged over two replicate measurements. We conducted *t*-tests to identify differentially expressed genes (DEGs). The DEGs with *p*-value < 0.05 and fold change >1.5 at more than 2 time points were selected. All the computations were performed in the MATLAB bioinformatics toolbox.

We downloaded the experimentally verified TF-TG interaction data from TFactS database [18]. This database includes interaction from TRED, TRDD, PAZAR, NFIregulomeDB databases and their own experimental predictions. This database provides ≈ 7000 interactions between 2700 TGs and 330 TFs. To test the algorithm’s performance on an independent topology data acquired elsewhere, we downloaded the TF-TG interaction data from HTRIdb database developed by Bovolenta et al. [19].

### Generation of synthetic data and network configurations

We created 100 different expression matrices for each case study, by randomly generating *A* and fixed *P* matrices according to Eq. 2. We used Gaussian distribution to generate random elements of *A* (both positive and negative values), while keeping its null space. We used Matlab function ^′^ *randn* ^′^ for this purpose. The different network configurations for EGF and HRG systems are generated as follows. First, we identified two NCA compliant sub-networks. Then, a subset of the components of each sub-network is randomly selected by randomly removing one or more TF, with their corresponding TGs. Then each of the new sub-networks are checked for NCA criteria. In this way, we generated 100 NCA compliant network configurations for each system.

### Statistical analysis and calculations

All the calculations were performed using Matlab R12 (Mathworks Inc.). The standard NCA algorithms (GNCA-r, FastNCA and ROBNCA) are downloaded from respective websites which are publicly available. The full ISNCA algorithm is available for download at the corresponding author’s website.

### Gene ontology analysis

The significantly enriched gene ontology terms or biological processes are identified using the GOrilla tool developed by Eden et al. [20].

## Results

**Fig. 2:**
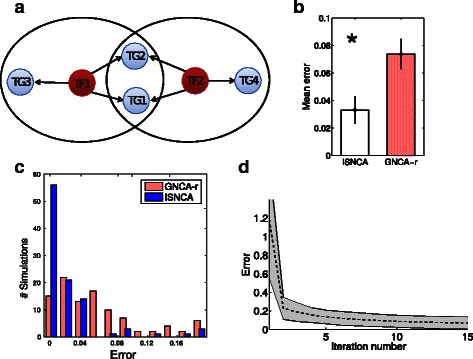
Synthetic, toy case-study and comparison between ISNCA and standard NCA. **a** The complete network is composed of four target genes (TGi, *gray*) and two transcription factors (TFi, *red*). The two sub-networks of the ISNCA are encircled (*solid line*) and share TG1 and TG2. **b** Comparison of the error between the ISNCA (*white*) and the GNCA-r (*red*). The mean error of 100 simulations (see ‘Methods’) of the ISNCA was significantly lower (*p* < 10^−12^, Kruskal-Wallis test) than of the GNCA-r. The error bars are the standard error of means (SEM). **c** The distribution of the error of the ISNCA (*blue*) and the GNCA-r (*red*). The errors of 91 % of the ISNCA simulations were under 0.04, compared to only 50 % of the GNCA-r. **d** The convergence of the ISNCA mean error (*n* = 100) was rapid, stabilized after 3–5 iterations. Shaded area represents standard deviation at iterations

We first tested the algorithm on a small toy network containing four TGs, two TFs and six interactions (Fig. 2 a). The gene expression matrix incorporated three time points, and the TF profiles of the network was reconstructed using Eq. 2 (see ‘Methods’). The complete network satisfies the NCA conditions, and can therefore be solved by NCA-based methods. We wanted to examine the accuracy of our iterative approach layered on a standard NCA method (GNCA-r) and compare it with the same standard GNCA-r method that solves the entire network. We generated 100 random initial *E* matrices and applied ISNCA and GNCA-r to reconstruct *A*, and *P*. The mean reconstruction error (see ‘Methods’) of the ISNCA method was significantly lower (*p* < 10^−12^, Kruskal-Wallis test; *n* = 100) compared with the GNCA-r (Fig. 2 b). The ISNCA yielded error of less than 0.04 in 91 % of the simulations (91/100), compared to 50 % (50/100) by the GNCA-r (Fig. 2 c). The reconstruction errors of 100 simulations converged after 3–5 iterations (Fig. 2 d) and stayed stable thereafter, with a sharp drop already after the second iteration.

**Fig. 3:**
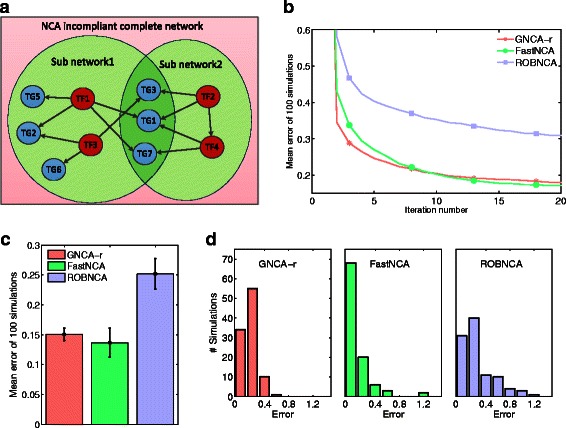
An incompliant synthetic network and the two iterating sub-networks. **a** The red outer boundary represents the complete network that does not satisfy the conditions for uniqueness of the solution. The two sub-networks are shaded in green are each compliant, and solved iteratively by the ISNCA, build on GNCA-r, FastNCA and ROBNCA. Note that TF4 is also a gene. **b** All the ISNCA procedures converged to a stable solution after about 10–15 iterations, with the first and second iterations have the strongest reduction. **c** Comparison of the mean error of 100 simulations for each ISNCA indicates low error for ISNCA (FastNCA) for small network sizes and **d** Error distributions of 100 ISNCA simulations, built on the three NCA algorithms. For this small network, mean error of the ISNCA with GNCA-r and FastNCA was significantly lower (*p* < 10^−4^, one-way ANOVA) than the ISNCA (ROBNCA)

We constructed a more complex example in which the entire network was in-compliant, i.e. the conditions that guarantee a unique solution up to a scale matrix were not satisfied (Fig. 3 a, red shaded). The common procedure of NCA based methods is then to reduce the network size, for instance by removing TF4 and its corresponding genes TG1 and TG7. We divided this complete network into two sub-networks (Fig. 3 a, green shaded), each with two TFs, with six genes in the first sub-network and 4 genes in the second. Notice that TF4 is also a target gene for TF2. Recall also that it is not possible to guarantee a unique solution to the entire network using standard NCA methods, so comparison of the ISNCA to these is not feasible. We tested the reconstruction of the complete network by the ISNCA, layered with three different NCA methods; GNCA-r, Fast NCA and ROBNCA (see ‘Methods’). All the three ISNCA layers converged to a stable solution with a sharp drop after 3–5 iterations (Fig. 3 b). The mean error of the ISNCA (GNCA-r) and the ISNCA (Fast-NCA) were significantly lower (*p* < 10^−4^, one-way ANOVA; *n* = 100) than the ISNCA (ROBNCA) (Fig. 3 c). ISNCA (FastNCA) was the most accurate approach for this network, with 68 % the simulations resulted in error of less than 0.001. In contrast, more than 69 % of the simulations by the ISNCA (ROBNCA) produced error larger than 0.1.

**Fig. 4:**
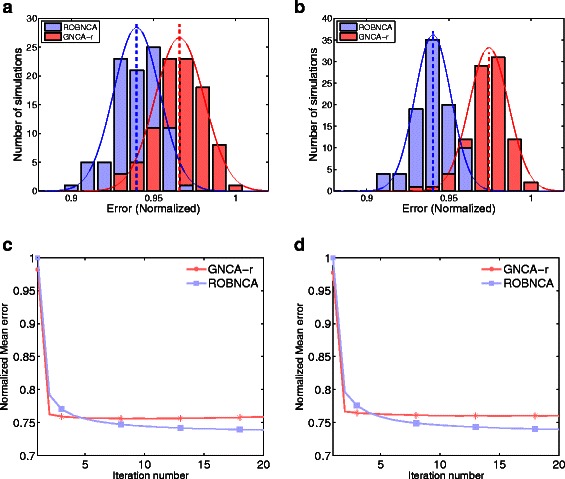
ISNCA applied on a real, large biological network. Comparison of the error distributions 100 generated networks of varying sizes (see Table 1 for details) with ISNCA algorithm built on two NCA methods, ROBNCA (*blue*) and GNCA-r (*red*) for EGF (**a**) and HRG (**b**) microarray data. The errors are normalized for comparison purposes. The mean error over 100 networks is presented as thick dashed lines for both algorithms, blue for ROBNCA and red for GNCA-r. c-d) The convergence properties of the ISNCA with ROBNCA (*blue*) and GNCA-r (*red*) algorithms for EGF (**c**) and HRG (**d**) data. The mean error over 100 networks was stabilized after 3–4 iterations for ISNCA with GNCA-r and after 10–15 iterations for ISNCA with ROBNCA

To test the ability of the ISNCA algorithms to reconstruct large, real biological networks, we finally used two microarray gene expression matrices for the epidermal growth factor (EGF) and heregulin (HRG) stimuli systems [17]. We generated (see ‘Methods’) 100 network configurations for each system, consisting of different sets of TFs and TGs based on the interaction data downloaded from TFactS database [18]. Each of the networks generated was relatively large (See Table 1 for network size comparison reconstructed by ISNCA and any NCA algorithm, GNCA-r) and initially (before the network reduction procedure), did not satisfy the conditions for uniqueness of the solution. We tested our iterative algorithm with two layouts, the GNCA-r and ROBNCA. The FastNCA algorithm can reconstruct network size with maximum TFs equal to number of experimental time points, and therefore could not be used to reconstruct these large networks. Reconstruction with ISNCA was successful in all the 200 trials. The ISNCA algorithm converged relatively fast, after about 5 iterations in all the 100 simulations tested for each EGF (Fig. 4 a) and HRG (Fig. 4 b) systems. We found that ISNCA (ROBNCA) performed better than the ISNCA (GNCA-r), with a lower mean error for both EGF (Fig. 4 c) and HRG (Fig. 4 d) systems.

**Table 1:** The network properties of 100 generated EGF and HRG networks. Here values are presented as range (minimum-maximum) of 100 networks

Network	EGF network		HRG network	
property	GNCA-r	ISNCA	GNCA-r	ISNCA
# of TGs	207–320	232–342	268–400	325–443
# of TFs	43–54	64	39–48	64
# of interactions	333–509	449–627	429–653	653–854

### Selection of delta and convergence properties

**Fig. 5:**
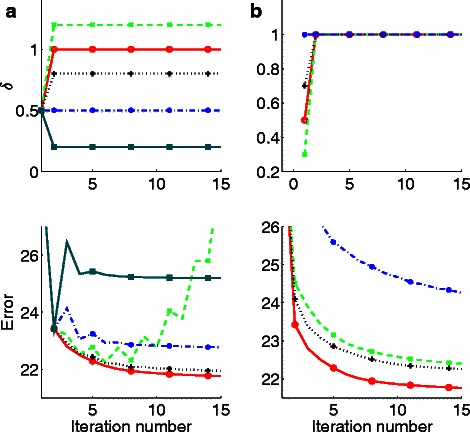
The attenuation factor, delta(*δ*) and ISNCA convergence properties. The selection of attenuation factor, *δ* with iterations is presented for five different scenarios (**a**) and four other scenarios (**b**). The corresponding two lower panels shows the error convergence responses to the ISNCA algorithm with ROBNCA, for the different *δ* values. **a** The *δ* is fixed at 0.5 at iteration one (k = 1) in all scenarios and *δ* is varied (0.2, 0.5, 0.8, 1.0 and 1.2) at subsequent iterations, k >1. The *δ* (k = 1) = 0.5 and *δ* (k >1) = 1.0 gives the best error and smooth convergence. b) The *δ* is fixed at 1.0 at all iterations (k >1) in all scenarios and *δ* is varied (0.3, 0.5, 0.7 and 1.0) at first iteration. The delta values *δ* (k = 1) = 0.5 and *δ* (k >1) = 1.0 gives the best error and smooth convergence (*red solid line*)

We also tested the convergence properties of the ISNCA algorithm as a function of the attenuation factor, *δ* (see ‘Methods’). We began *δ* with a fixed value in the first iteration (*k* = 1), and changed this value at the second iteration and onwards (Fig. 5 a upper panel). We found that the response to the values *δ* (*k* = 1) = 0.5 and *δ*(*k* >1) = 1 was a stable and fast convergence compared with the other functions (Fig. 5 a lower panel; see also Figure S1 in the Additional file 1). Of the functions we tested, only values of *δ* larger than 1 resulted in divergence. We found that the value of *δ* at the first iteration is also important, with 0.5 being the optimal value (Fig. 5 b upper panel, red) yielding best convergence (Fig. 5 b lower panel, red). Other increasing or decreasing values *δ* of with iteration were found to yield non-optimal convergence properties (see Additional file 1: Figure S1).

## Discussion

Reconstruction of complex transcriptional networks from expression data is a common approach that helps to understand cellular signaling and gene regulation. These reconstructions by existing NCA algorithms are limited to relatively modest network sizes because of the necessary criteria (See ‘Methods’). The most common reduction procedure to satisfy the criteria is the pruning approach [10]. In our case, it removed up to 34 % (224/653) of the network connections, and almost 40 % (25/64) of the initial TFs (Table 1). The ISNCA approach reconstructed relatively the larger networks in terms of number of TFs, TGs and interactions between them compared to standard NCA algorithms (Table 1). We repeated the same analysis, comparing network sizes reconstructed by ISNCA approach with standard NCA algorithms, but this time using an independent TF-TG topology, acquired from HTRIdb (see ‘Methods’) [19]. The ISNCA algorithm demonstrated again superior performance to the current NCA algorithm (see Table S1 in Additional file 1), indicating that ISNCA performance is not limited by the quality of the TF-TG interactions.

There is no direct manner to control which TFs are pruned, and potentially removing TFs that may be important for a specific study. To demonstrate the consequences of this, we tested and compared the standard NCA algorithm with the ISNCA using 100 network configurations and microarray data obtained from breast cancer cells treated with EGF (Fig. 4). Firstly, the transcription factor FOXA1 (forkhead box protein A1) that is known to be strongly involved in breast cancer [21–23] was removed by the NCA algorithm in 84 % (84/100) of the configurations (it was retained 100 % by the ISNCA), loosing the ability to study its effect on the network. This occured despite the importance of FOXA1 in process involved in cancer development: it forms a strong network with ER- *α* (estrogen receptor-alpha) and GATA-3 (GATA binding protein 3) and controls the gene expression pattern in luminal subtype A breast cancers [21]. In addition, it is shown to be a potential prognosis marker and a significant predictor of good outcome in breast cancer [23]. Secondly, the activating transcription factor 2 (ATF2) is also strongly involved in breast cancer studies [24–26] and was removed by the NCA prior to the analysis. The active ATF2 regulates the genes MMP-2 and MMP-9 in the transforming growth factor (TGF- *β*) induced MCF10A human breast epithelial cells, and induces migration and invasion of MCF10A cells [26]. Additionally, ATF2 regulates the transcription of maspin and GADD45- *α* genes in mammary tumors [25]. Thirdly, ATF3 is known to be strongly involved as both tumor suppressor and an oncogene in breast cancer cells, and was proposed as potential therapeutic target in breast cancer treatment [27–29]. ATF3 up-regulates the genes TWIST1, fibronectin (FN)-1, SNAIL and SLUG in MCF10A cancer cells [27]. Together with FOXA1 and ATF2, ATF3 was completely removed from the network, reducing the possibility that these regulators could be analyzed and targeted. Similarly, many other pivotal TFs in breast cancer were removed by NCA (Table 2) but retained by ISNCA, which not only reconstructed large gene regulatory networks, but also retained their key components.

**Table 2:** The list of transcription factors removed by standard NCA algorithm but retained by ISNCA in the EGF data set and its involvement in previous breast cancer studies

TF symbol	Description	Breast cancer related?	% removed	PMIDs
ATF2	Activating transcription factor 2	Yes	92	19331149, 18677098,
				17079470, 17258390
FOXA1	Forkhead box A1	Yes	84	19261198, 24528009,
				25155268, 17373880
SMAD5	SMAD family member 5	Yes	91	23334326, 19096363
				17786313
RARG	Retinoic acid receptor-gamma	Yes	91	21482774, 15375546
				10928067
REL	v-rel avian reticuloendotheliosis viral oncogene	Yes	13	21482774, 15375546
ATF3	Activating transcription factor 3	Yes	91	23921126, 24494067,
				20930144, 17952119
ETV4	ETS translocation variant 4	Yes	89	17467662, 22075993
FOS	FBJ murine osteosarcoma viral	Yes	84	15319566, 19925682
	oncogene homolog			
FOSL1	Fos-related antigen 1	Yes	93	19925682, 21570421
JUNB	jun B proto-oncogene	Yes	94	24073962, 8417822
MYBL2	Myb-related protein B	Yes	83	25337223, 25502082,
				22037783
NFIA	Nuclear factor 1 A-type	Yes	90	24393253, 20525248
NOTCH1	Notch homolog 1,	Yes	81	25287362, 25544568,
	translocation associated			24970818
PAX3	Paired box 3	Yes	89	24438019, 20525248
SMAD7	SMAD family member 7	Yes	82	22841502, 22033246
FLI1	Fli-1 proto-oncogene,	Yes	79	25379017, 17727680,
	ETS transcription factor			17172821

% removed indicates that number of times standard NCA failed to retain the particular transcription factor on 100 tested networks. PMID is the PubMed database identification number

We repeated the same analysis on an independent microarray data set in order to demonstrate the biological importance of the ISNCA and its implications on cancer studies. Here we analyzed the data set obtained from breast cancer cells treated with HRG (see ‘Methods’ and Table 3). The ISNCA persistently retained several key TF that we suspected were relevant to the breast cancer studies, whereas these TF were removed by other standard NCA algorithms. By closer examination of the TGs which are regulated by those TFs, we found (Table 4 and Additional file 1: Table S2) that they are strongly involved in biological processes relevant to breast cancer studies. What appears to be a simple pruning of several TFs by the standard NCA algorithms (consequentially eliminates their corresponding TGs) may impair our analysis of the data, and weaken our understanding of the processes involved in cancer.

**Table 3:** The list of transcription factors removed by standard NCA algorithm but retained by ISNCA in the HRG data set and its involvement in previous breast cancer studies

TF symbol	Description	Breast cancer related?	% removed	PMIDs
ATF2	Activating transcription factor 2	Yes	81	19331149, 18677098,
				17079470, 17258390
FOXA1	Forkhead box A1	Yes	85	19261198, 24528009,
				25155268, 17373880
SMAD5	SMAD family member 5	Yes	86	23334326, 19096363
				17786313
RARG	Retinoic acid receptor-gamma	Yes	81	21482774, 15375546
				10928067
REL	v-rel avian reticuloendotheliosis viral oncogene homolog	Yes	71	21482774, 15375546
E2F2	E2F transcription factor 2	Yes	92	25028721, 24934442,
				24362522
NFkB2	nuclear factor of kappa light polypeptide gene enhancer in B-cells 2	Yes	89	12835724, 7478612
TCF7	transcription factor 7	Yes	91	26079538, 24401947
SP2	Sp2 transcription factor	Yes	93	20382698
PAX6	Paired box 6	Yes	86	21944253, 25323813
PGR	Progesterone receptor	Yes	91	26277479, 26153859

% removed indicates that number of times standard NCA failed to retain the particular transcription factor on 100 tested networks. PMID is the PubMed database identification number

**Table 4:** Significantly enriched gene ontology terms/biological processes in the genes regulated by transcription factors in Table 2 from EGF data set

GO term	Description	*P*-value
GO:0030154	cell differentiation	8.97E-04
GO:0008284	positive regulation of cell proliferation	2.00E-03
GO:0048869	cellular developmental process	4.38E-03
GO:0010557	positive regulation of macromolecule biosynthetic process	6.26E-03
GO:0031325	positive regulation of cellular metabolic process	6.06E-03
GO:0007219	Notch signaling pathway	8.35E-03
GO:0034097	response to cytokine	8.49E-03
GO:0048583	regulation of response to stimulus	8.31E-03
GO:0000904	cell morphogenesis involved in differentiation	1.65E-02
GO:0006935	chemotaxis	1.62E-02
GO:0001525	angiogenesis	1.59E-02
GO:0000902	cell morphogenesis	2.07E-02
GO:0030334	regulation of cell migration	2.92E-02
GO:0038061	NF-kappaB signaling	3.49E-02

^*^ *P*-values are FDR adjusted

In addition to the downsizing the network, the original NCA criteria seem very harsh from biological perspective. Condition I of full-column rank on connectivity matrix *A*, means that there cannot be two or more TFs or TF combinations with the same regulatory functionality (see ‘Methods’). Condition III of full-row rank on TF activity matrix, *P* implies that there cannot be two or more TFs or TF combinations with the same temporal behavior. Both restrictions produce conservative solutions that are not always acceptable in biological processes. Our approach avoids these restrictions. Contrary to solving overlapping sub-networks independently [13], our ISNCA algorithm links together the sub-networks by predicting and updating the contribution of the common components at each iteration, and minimizes the error reconstruction of the entire network. We tested both approaches using a large number (>200) of network configurations using several independent systems (see ‘Methods’). The advantage of predict-update process became apparent from the analysis of both iterating and non-iterating, overlapping sub-networks. Firstly, we studied small synthetic network, where the reconstructed profiles could be compared to the original profiles (see ‘Methods’). The accuracy (Pearson’s correlation) of the ISNCA was significantly (*p* < 10^−4^, two-samples *t*-test) higher than the one computed from independent networks (Additional file 1: Figure S2), for all the four TFs. Secondly, we analyzed two large biological networks (EGF and HRG systems), each subdivided to two sub-networks. We repeated the procedure with 100 different network structures for each system. For all large systems we tested, the mean of the reconstruction error was significantly (*p* < 10^−10^, two sample *t*-test, *n* = 100) lower (about 25 % reduction) for ISNCA algorithm than independent networks approach (Additional file 1: Figure S3). Thirdly, we compared the correlation (Pearson) of the reconstructed profiles of the TF that were shared between the two subnetworks, to evaluate the consistency of the reconstruction. This analysis also confirmed that the TF profiles calculated by the ISNCA were more accurate than the ones calculated from independent networks (Additional file 1: Figure S4). We stress that similarly to other NCA methods, the quality of the TF profile reconstruction depends on the noise and quality of the expression data. Together, the analysis confirms that the link between sub-networks is necessary to obtain more accurate (in terms of low reconstruction error and consistent TF profiles) and feasible network reconstruction. The predict and update feature of ISNCA algorithm is comparable to nonlinear iterative partial least squares (NIPALS) algorithm used for PCA and PLSR modeling [30]. In the NIPALS, the score matrix (equivelant to our A matrix) is predicted and updated until it reaches a desired convergence criteria.

We did not focus here on the optimal division of the complete network into NCA compliant ones. We initially assumed a certain set of TGs are shared between the sub-networks. In this work, we divided the main network heuristically, with the only requirement that both sub networks are compliant, so that they can be solved by a standard NCA method. The number of common components, and their interconnections, will ultimately affect the solution. In practical terms, it is possible to apply the algorithms that choose the common components and predict the optimal configuration. One heuristic approach [13, 31] generated overlapping sub-networks which satisfy NCA compliancy criteria. This approach starts with a randomly chosen sub-network composed of several TFs. If this sub-network is not compliant, it removes a set of TFs that did not satisfy the NCA criteria, and substitute with the new set of TFs. This process is repeated until it finds an NCA compliant sub network. Another approach proposed [32] finds the best network structure, A, which satisfies the NCA conditions. Here, several NCA compliant network structures are generated in an intelligent manner, based on mixed integer, non-linear programming optimization formulation. It then checks the reconstruction error of all generated networks and chooses the network with the minimum error. The Branch and Bound algorithms that are implemented to solve NP-hard discrete optimization problems can also be used to identify the best network configurations [33]. It can do so by either minimizing or maximizing the number of common components (TGs, TFs, interactions), or focus on a search to minimize the error of the entire network. The former case does not require running ISNCA at every iteration, only to test the network configurations for NCA compliancy, and is a faster and easier problem to solve than the latter. Several modeling approaches are developed for network divisions, finding modularizations, with specific constraints based on Branch and Bound formulations. For instance, a branch and bound based approach to divide a given cellular network into several, smaller sub-networks or modules [34] or the partitioning the acyclic networks into disjoint subnetworks [35]. It is possible to combine these approaches with NCA criteria as constraints for finding the optimum NCA compliant sub-networks and ISNCA for the best feasible reconstruction. Additionally, we proposed here network configurations sharing only TGs, but it can be easily extended to include also TFs as common components. We provide solutions to this problem formulation in the Additional file 1: Supplementary information and Additional file 1: Figure S5. However, since the ISNCA converged in all the network configurations we tested, it was not necessary to include TFs as common sub-networks components.

The ISNCA can be further expanded to reconstruct extensively large network in a recursive manner. With the recursive approach, we divide the network into compliant or non- compliant sub-networks. These can be further divided until each sub-network is compliant (see Additional file 1: Figure S6). The algorithm works recursively at each iteration between the parents sub networks, the recursive ISNCA iterates between the children sub-networks until convergence to a local solution. This solution is sent to the next iteration in the parents sub networks. We can subdivide the entire network to 2n sub-networks with n is the number of generations, all the generations but the last may be incompliant. In this manner, we are able to find a local solution to any large network. Theoretically, n can be arbitrarily large, but the computation complexity is also increased exponentially. The iterative ISNCA is subjected to our future work.

All the networks we tested (>400) demonstrated rapid convergence. We found that the convergence was also dependent on the parameter *δ* the attenuation factor that weights the update of the common expression matrix in the next iteration (see Eq. 10 in ‘Methods’). We tested different variations of *δ* and found (heuristically) that the convergence of the ISNCA was optimal when the algorithm applies the weight of *δ*= 0.5 at the first update, followed by consequent updates of *δ*= 1 (Fig. 5). We stress that *δ*= 0 transforms the problem to the simple network division with no updates (independent networks), discussed above. Similarly to the known NIPALS algoritms, convergence cannot be proven in general [36], and is dependent on the topology and the network division. However, similarly to convergence of NIPALS [30], ISNCA was found to converged in parctice (it converged in the houndreds of simulations and network configurations we tested).

## Conclusions

Taken together, we developed an iterative approach, which employs existing NCA algorithms to solve iteratively networks without reducing their size. The ISNCA is able to i) incorporate these known properties of redundancy and cooperative behavior of TFs, making the solution more biological plausible, and ii) prevent undesired elimination of potentially essential components, and iii) increase the size of the solution, incorporating more information into the network. We propose to apply our algorithm to study data obtained from any biological system.

## Availability of supporting data

Data supporting the results were downloaded from GEO database, array express accession number: GSE13009 [17]. The TF-TG interaction data was downloaded from TFactS database [18] and HTRIdb database [19].

## Additional file

Additional file 1
**The supplementary files include an ISNCA formulation for the case where TFs are also shared between the seb-networks.** We also included Figures S1-S6 that evaluate the performance of the ISNCA and add extended description. (PDF 394 kb)

## Abbreviations

ATF: Activating transcription factor
NIPALS: Nonlinear iterative partial least squares
DEG: Differentially expressed genes
PCA: Principle component analysis
EGF: Epidermal growth factor
PLSR: Partial least squares regression
GNCA-r: General NCA
ROBNCA: Robust NCA
HRG: Heregulin
SVD: Singular value decomposition
ICA: Independent component analysis
TF: Transcription Factor
ISNCA: Iterative sub-network component analysis
TG: Target gene
NCA: Network component analysis
