# Supplementary material for: Iterative sub-network component analysis enables reconstruction of large scale genetic networks
Source: BMC Bioinformatics. 2015 Nov 4;16:366. doi: 10.1186/s12859-015-0768-9 (PMC4634733; doi:10.1186/s12859-015-0768-9)
Supplement: Additional file 1 — The supplementary files include an ISNCA formulation for the case where TFs are also shared between the seb-networks. We also included Figures S1-S6 that evaluate the performance of the ISNCA and add extended description. (PDF 394 kb) [file 12859_2015_768_MOESM1_ESM.pdf]

ISNCA formulation for shared TFs and TGs

The current proposed algorithm, the iterative sub-network component analysis (ISNCA) is extended easily to solve the NCA compliant sub-networks that share common TFs and TGs. In order to apply the ISNCA algorithm, we first divide the network into two compliant sub-networks. The expression and connectivity matrices for each sub-network can be represented by

$$E_1 = \begin{bmatrix} E_{u1} \\ E_c \end{bmatrix}, E_2 = \begin{bmatrix} E_{u2} \\ E_c \end{bmatrix} \tag{1}$$

and

$$A_1 = \begin{bmatrix} A_{uu1} & A_{uc1} \\ A_{cu1} & A_{ccc} \end{bmatrix}, A_2 = \begin{bmatrix} A_{ccc} & A_{cu2} \\ A_{uc2} & A_{uu2} \end{bmatrix} \tag{2}$$

with $E_{ui} \in \mathbb{R}^{nui \times m}$ and $E_c \in \mathbb{R}^{nc \times m}$ denote the expression matrices of sub-networks $i = 1, 2$. $A_{uui} \in \mathbb{R}^{nui \times lui}$, $A_{cui} \in \mathbb{R}^{nc \times lui}$, $A_{uci} \in \mathbb{R}^{nci \times lc}$, and $A_{ccc} \in \mathbb{R}^{nc \times lc}$ are the partition matrices of A, of sub network $i = 1, 2$, the subscript indices u, c denotes the unique and common components of the sub networks. The first, second and third subscript indices of any partition matrices denote TGs, TFs and sub-networks respectively. In all the following, when we write A_i, E_i or P_i, we refer to matrices of the entire sub-network i, including both its exclusive and common components.

The entire network can be described in the following manner:

$$A = \begin{bmatrix} A_{uu1} & A_{uc1} & \mathbf{O}_2 \\ A_{cu1} & A_{ccc} & A_{cu2} \\ \mathbf{O}_1 & A_{uc2} & A_{uu2} \end{bmatrix} \tag{3}$$

The matrices $\mathbf{O}_1 \in \mathbb{R}^{nu2 \times lu1}$ and $\mathbf{O}_2 \in \mathbb{R}^{nu1 \times lu2}$ denote zero matrices. The corresponding partitions of E and P are obtained as follows:

$$E = \begin{bmatrix} E_{u1} \\ E_c \\ E_{u2} \end{bmatrix}, \qquad P = \begin{bmatrix} P_{u1} \\ P_c \\ P_{u2} \end{bmatrix} \tag{4}$$

where, $P_{ui} \in \mathbb{R}^{lui \times m}$, $P_c \in \mathbb{R}^{lc \times m}$ are the activities of unique and common TFs of sub-network i respectively. Note that P contains both unique and common components.

Example 1. *Network decomposition: Consider the network presented in the supplementary Figure **S3**). The connectivity matrix A can be decomposed to the exclusive components and the common components in the following*

manner:

$$A = \begin{bmatrix} A_{uu1} & A_{uc1} & \mathbf{O}_2 \\ A_{cu1} & A_{ccc} & A_{cu2} \\ \mathbf{O}_1 & A_{uc2} & A_{uu2} \end{bmatrix} = \left[\begin{array}{c|c|c|cc} & tf_1 & tf_3 & tf_2 & tf_4 \\ tg_5 & 1 & 0 & 0 & 0 \\ \hline tg_1 & 1 & 0 & 1 & 1 \\ tg_2 & 1 & 1 & 0 & 0 \\ tg_3 & 0 & 1 & 1 & 0 \\ tg_6 & 0 & 1 & 0 & 0 \\ tg_7 & 1 & 0 & 0 & 1 \\ \hline tf_4 & 0 & 0 & 1 & 0 \end{array}\right] \tag{5}$$

and partition matrices for sub-networks 1 and 2 respectively are,

$$A_{uu1} = \begin{bmatrix} 1 \end{bmatrix}, \quad A_{uc1} = \begin{bmatrix} 0 \end{bmatrix}, \quad A_{cu1} = \begin{bmatrix} 0 \\ 1 \\ 1 \\ 0 \\ 0 \end{bmatrix}, \quad A_{ccc} = \begin{bmatrix} 1 \\ 1 \\ 0 \\ 1 \\ 1 \end{bmatrix}, \quad \mathbf{O}_1 = \begin{bmatrix} 0 \end{bmatrix} \tag{6}$$

$$A_{uu2} = \begin{bmatrix} 1 & 0 \end{bmatrix}, \quad A_{uc2} = \begin{bmatrix} 0 \end{bmatrix}, \quad A_{cu2} = \begin{bmatrix} 1 & 1 \\ 0 & 0 \\ 1 & 0 \\ 0 & 0 \\ 0 & 1 \end{bmatrix}, \quad A_{ccc} = \begin{bmatrix} 1 \\ 1 \\ 0 \\ 1 \\ 1 \end{bmatrix}, \quad \mathbf{O}_2 = \begin{bmatrix} 0 & 0 \end{bmatrix} \tag{7}$$

To initialize the ISNCA algorithm, we divide the expression matrix, E to E_i using equation 1 and connectivity matrix, A to A_i using equation 2. At the start of each iteration k, we compute solution to $\|E_i(k) - A_iP_i\|$, separately for sub-networks 1 and 2 using any standard NCA method, and obtain $\hat{A}_i(k)$ and $\hat{P}_{ui}(k)$. We can then proceed to construct $\hat{A}(k)$ and $\hat{P}(k)$ by combining equations 2 and 3, as

$$\hat{A}(k) = \begin{bmatrix} \hat{A}_{uu1} & \hat{A}_{uc1} & \mathbf{O}_2 \\ \hat{A}_{cu1} & \hat{A}_{ccc} & \hat{A}_{cu2} \\ \mathbf{O}_1 & \hat{A}_{uc2} & \hat{A}_{uu2} \end{bmatrix}, \hat{P}(k) = \begin{bmatrix} \hat{P}_{u1}(k) \\ \hat{P}_c \\ \hat{P}_{u2}(k) \end{bmatrix} \tag{8}$$

and calculate the error of the entire network,

$$e(k) = \left\| E - \hat{A}\hat{P} \right\|_F \tag{9}$$

Here, $\hat{A}_{ccc}$ and $\hat{P}_c$ are calculated from both sub networks 1 and 2. Therefore it is important to choose the best contribution either from sub network 1 or 2 or average of 1 and 2 based on the lowest error of reconstruction according to equation 9. If the error does not converge (see below), we proceed to update the sub-networks in the following manner. Let $T_i(k)$ be the common TGs contribution from sub-networks i, that is,

$$T_1(k) = \hat{A}_{cc1}(k)\hat{P}_{c1}(k), \qquad T_2(k) = \hat{A}_{cc2}(k)\hat{P}_{c2}(k) \tag{10}$$

We then update the matrices E_1 and E_2 for next iteration, from equation 11 by subtracting the common TGs contribution from other sub-network, that is,

$$E_1(k+1) = \begin{bmatrix} E_{u1} \\ E_c - \delta \cdot T_2(k) \end{bmatrix}, \qquad E_2(k+1) = \begin{bmatrix} E_{u2} \\ E_c - \delta \cdot T_1(k) \end{bmatrix} \tag{11}$$

Here, $\delta \in [0, 1]$ denotes the attenuation factor (see below for details). Notice that E_c and E_{ui} do not change from iteration to iteration as they represent the original expression matrices. We then proceed to the next iteration and predict the solution to the expression $\|E_i(k+1) - A_i P_i\|$ using standard NCA methods. We keep iterating until the reconstruction error in equation 9 for the entire network is sufficiently small, for instance by

$$e(k+1) - e(k) < \epsilon \tag{12}$$

In simulations, we can set ϵ to be 1e-05 and maximum number of iterations to 100.
